# Supplementary material for: Evaluation of Semen Self-Sampling Yield Predictors and CTC Isolation by Multi-Color Flow Cytometry for Liquid Biopsy of Localized Prostate Cancer
Source: Cancers (Basel). 2023 May 9;15(10):2666. doi: 10.3390/cancers15102666 (PMC10216665; doi:10.3390/cancers15102666)
Supplement: Supplementary file 1 [file cancers-15-02666-s001.zip › SupplementaryInformation/Figure S1.pdf]

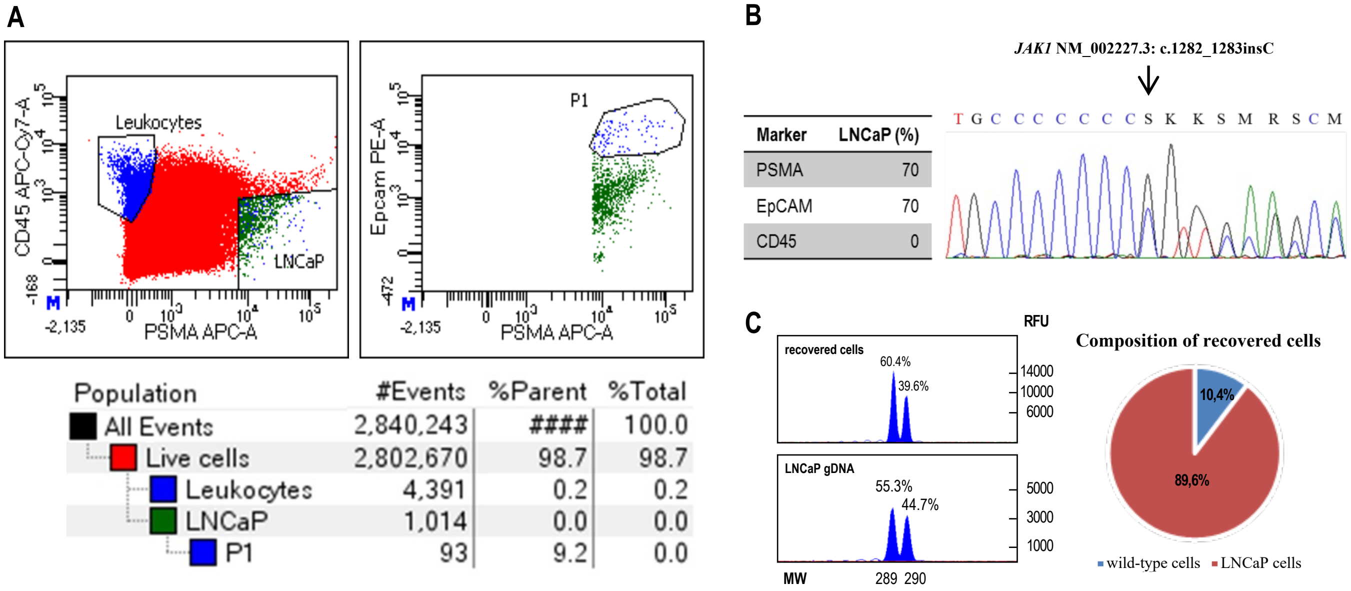

Figure S1. Spike-in approach for the setting up of multi-color FACS sorting of PCa cells from semen.
Semen samples were obtained from healthy donors and liquefied at RT for 30 mins. LNCaP, a human prostate adenocarcinoma cell line cells carrying a specific single-nucleotide insertion within exon 9 of the *JAK1* gene (NM_002227.3: c.1282_1283insC), were spiked-in at different amount (10, 100, 1000) in 1 mL of semen. Each sample was then centrifuged at 450 g at room temperature for 10 minutes to separate the cellular component from the seminal plasma. The resulting cell pellet was stained, and positive cells were sorted with FACSAria III (BD Biosciences, San Jose, USA). The purity of recovered cells was estimated using a PCR-based assay targeting the LNCaP-specific *JAK1* mutation.
A. Example of gate determination to identify and sort 100 LNCaP cells (Syto16+/7AAD-/PSMA+/CD45-/EpCAM+) spiked in the semen of a healthy donor. The cytograms show CD45 negative/PSMA positive cells (LNCaP gate) and PSMA-EpCAM positive cells (gate P1), accounting for 9.2% of CD45 negative/PSMA positive cells.
B. On the left, cell-surface molecule profile of the LNCaP cell line: the percentage of positive cells is shown for each of the marker tested. On the right, electropherogram showing the *JAK1* NM_002227.3: c.1282_1283insC in the heterozygous state in LNCaP cells.
C. Fluorescent PCR assay, amplifying *JAK1* exon 9, was performed on a fraction (1/3) of the 93 positive cells sorted (recovered cells; upper panel) and on genomic DNA from LNCaP cells (LNCaP gDNA; lower panel), used as control. On the left, blue peaks correspond to the PCR-labeled products, whose relative quantitation is reported (%). MW: molecular weight, as assessed by the size standard (ROX-500 HD, not shown); RFU: relative fluorescence units. On the right, the pie chart shows the percentage (89.6%) of the cells recovered by FACS, as assessed by the fluorescent PCR assay.
